# Sterol targeting drugs reveal life cycle stage-specific differences in trypanosome lipid rafts

**DOI:** 10.1038/s41598-017-08770-9

**Published:** 2017-08-22

**Authors:** Aabha I. Sharma, Cheryl L. Olson, João I. Mamede, Felipe Gazos-Lopes, Conrad L. Epting, Igor C. Almeida, David M. Engman

**Affiliations:** 1 Department of Pathology, Northwestern University, Chicago, Illinois USA; 2 Department of Microbiology-Immunology, Northwestern University, Chicago, Illinois USA; 3 Department of Cell and Molecular Biology, Northwestern University, Chicago, Illinois USA; 4 Department of Pediatrics, Northwestern University, Chicago, Illinois USA; 5 Department of Biological Sciences, University of Texas at El Paso, El Paso, TX USA; 6 Department of Pathology and Laboratory Medicine, Cedars-Sinai Medical Center, Los Angeles, CA USA

## Abstract

Cilia play important roles in cell signaling, facilitated by the unique lipid environment of a ciliary membrane containing high concentrations of sterol-rich lipid rafts. The African trypanosome *Trypanosoma brucei* is a single-celled eukaryote with a single cilium/flagellum. We tested whether flagellar sterol enrichment results from selective flagellar partitioning of specific sterol species or from general enrichment of all sterols. While all sterols are enriched in the flagellum, cholesterol is especially enriched. *T. brucei* cycles between its mammalian host (bloodstream cell), in which it scavenges cholesterol, and its tsetse fly host (procyclic cell), in which it both scavenges cholesterol and synthesizes ergosterol. We wondered whether the insect and mammalian life cycle stages possess chemically different lipid rafts due to different sterol utilization. Treatment of bloodstream parasites with cholesterol-specific methyl-β-cyclodextrin disrupts both membrane liquid order and localization of a raft-associated ciliary membrane calcium sensor. Treatment with ergosterol-specific amphotericin B does not. The opposite results were observed with ergosterol-rich procyclic cells. Further, these agents have opposite effects on flagellar sterol enrichment and cell metabolism in the two life cycle stages. These findings illuminate differences in the lipid rafts of an organism employing life cycle-specific sterols and have implications for treatment.

## Introduction

All eukaryotic cilia, whether motile (flagella), sensory or primary, share a highly conserved structure and serve as environmental sensors in a wide range of organisms and cell types, from protists to humans^1–4^. The best studied human ciliary sensors are the sensory receptors underlying our five senses—vision, taste, smell, hearing and touch^1, 5–8^. Ciliary sensory function is facilitated by the unique lipid environment of the ciliary membrane, which displays high membrane liquid order^4, 9–16^. These areas of liquid order reflect the presence of lipid rafts, microdomains which contain high levels 3-β-hydroxysterols, sphingolipids and proteins frequently having acyl modifications such as myristoylation or palmitoylation^9, 11, 12, 17^. Lipid rafts provide platforms for the assembly of cell signaling complexes in eukaryotes^12, 18, 19^.

Trypanosomes are single-celled eukaryotic pathogens that are transmitted by insects and cause a variety of diseases throughout the world. *Trypanosoma brucei*, the protozoan parasite that causes African sleeping sickness, is transmitted to humans and other animals by the bite of the tsetse fly. Like other pathogens transmitted by insects, *T. brucei* has evolved to adapt to life in different hosts. Two life cycle stages of *T. brucei* that are easily cultured in the laboratory are the procyclic form, found in the tsetse midgut, and the bloodstream form, which divides in the bloodstream of the mammalian host. Because of its genetic manipulability and clearly delineated flagellar structures, *T. brucei* has emerged as an excellent model organism for the study of the ciliary structure and function, membrane biogenesis and environmental sensing^20–23^.

Lipid rafts have been studied in *T. brucei* ^24, 25^ as well as in the related trypanosomes *Leishmania* ^26^ and *T. cruzi* ^27, 28^. Intracellular trypanosomatids *T. cruzi* and *Leishmania spp*. exploit lipid rafts for host immune evasion and infection^29, 30^. Work done by our laboratory and others have shown the flagellar membrane of *T. brucei* to be enriched in 3 β–hydroxysterols, sphingolipids, dually acylated proteins, and phospholipids^9, 31, 32^. GPI-anchored proteins such as Gp63, variant surface glycoprotein in bloodstream cells and procyclin in procyclic cells, that account for majority of the surface proteins in *T. brucei*, are not lipid raft associated^9, 33^ due to the unusual acyl and alkyl chain composition of the GPI anchor^33^.

Sterols are highly enriched in the flagellar lipid rafts of *T. brucei*, and these sterols are required for the flagellar membrane localization of proteins such as the myristoylated-palmitoylated calflagin proteins^9^. Cholesterol depletion of bloodstream cells via treatment with methyl-β-cyclodextrin (MBCD) prevents the proper localization of calflagin; MBCD has similar effects on other plant and animal cells^34–36^. *T. brucei* can synthesize and scavenge sterols and does so differently depending on the life cycle stage^37^. Procyclic cells synthesize sterols *de novo* and produce 24 β-alkyl sterols typical of fungi and other protozoans^38, 39^. They also scavenge cholesterol from the bloodmeal the tsetse consumes from its mammalian hosts. Bloodstream cells scavenge cholesterol, which is in abundance in the mammalian bloodstream. In both procyclic and bloodstream cells, cholesterol scavenging is believed to occur through receptor-mediated uptake^37^. As a result, the sterol in bloodstream cells is ~96% cholesterol, whereas the sterol in procyclic cells cultured *in vitro* contain approximately 60% scavenged cholesterol and the rest derivatives of ergosterol and cholesterol^40–42^.

In this work we explored two questions. The first is whether flagellar sterol enrichment responsible for the high membrane liquid order and lipid raft content is due to selective partitioning of one or more specific sterol species or to a general enrichment of all sterols equally. The second is whether *T. brucei* procyclic and bloodstream life cycle stages have different kinds of lipid rafts based on their having different sterol compositions. Our findings have implications for flagellar lipid raft biogenesis and are relevant to drug discovery strategies for development of agents to treat infections with eukaryotic pathogens having insect and mammalian life cycle stages.

## Results

### The trypanosome flagellar membrane has a sterol composition that is similar to, but more concentrated than, the cell body

**Figure 1:**
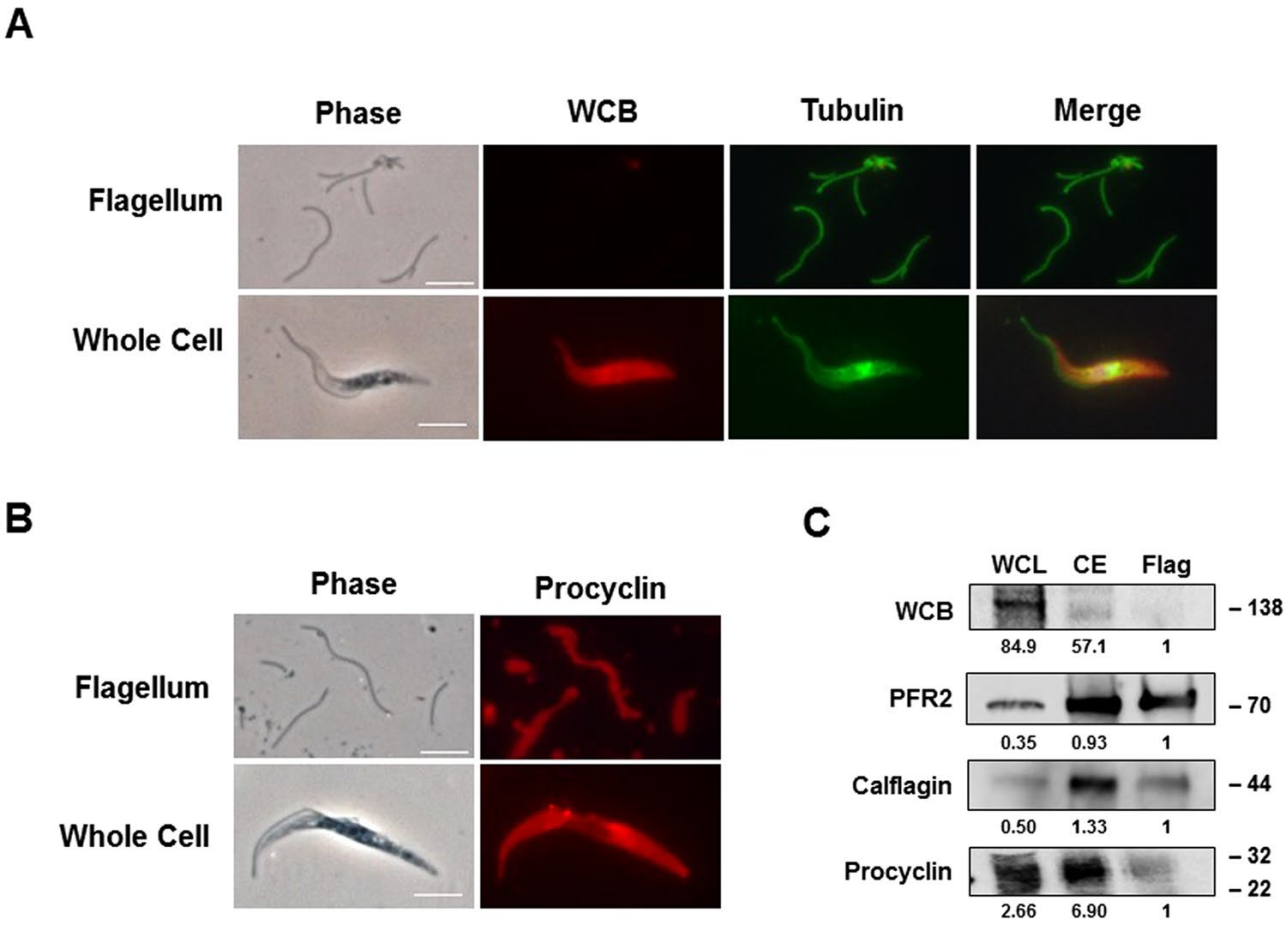
Analysis of purified flagella. Flagella were purified from *T. brucei* procyclic cells as described in Methods. (**A** and **B**) Immunofluorescence microscopy of purified flagella and whole cells using antibodies specific for tubulin (cytoskeleton) and procyclin (surface), and whole cell body (WCB) protein (cell bodies). Scale bar = 5 μm. (**C**) Western blot analysis of a whole cell lysate (WCL), a crude extract (CE) and purified flagella (Flag) using antisera specific for WCB (cell bodies), procyclin (surface), calflagin (flagellum) and PFR2 (flagellum). Relative protein levels normalized to the flagellar signal by densitometry are shown beneath each band. Numbers on the right represent molecular weight markers﻿ (in kDa).

Lipid rafts are highly enriched in cilia^9^ as well as the apical domains of polarized cells^43, 44^. In trypanosomes, the motile cilium (flagellum) -enriched molecules include sterols, sphingolipids, gangliosides, and some dually acylated proteins^9^. The lipid modifications on these proteins are required for association with lipid rafts^9, 45, 46^. What was not clear from previous studies is whether the flagellar enrichment of sterols, the key component of lipid rafts, is due to selective concentration of one or more individual sterol species, or general enrichment of all sterols found in the cell body membrane. In bloodstream cells, nearly all of the sterol is cholesterol, scavenged from the mammalian bloodstream^40, 41^. We therefore examined the sterol compositions of purified flagella and whole cells from procyclic cells, which possess multiple sterol species^40, 41^. The trypanosome flagellum is attached to the cell body along the length of the cell by a desmosome-like junction and the process of removing the flagellum by sonication obliterates the cell body. Therefore, we used whole cells rather than cell bodies for sterol extraction and computed cell body specific sterol content by subtracting flagellar sterol content from the whole cell content. We purified flagella from *T. brucei* procyclic cells by mild sonication and gradient fractionation (see Methods). The purity of the flagellar fractions was assessed by immunofluorescence microscopy and/or western blotting using markers for the cell body (WCB), flagellum (calflagin, paraflagellar rod protein 2 (PFR2)), or both cell body and flagellum (tubulin, cytoskeleton; procyclin cell/ciliary surface) (Fig. 1). This analysis showed that purified flagella are essentially free of cell body components and also that they contain both cytoskeletal (tubulin) and membrane (procyclin) elements of the flagellum.

**Figure 2:**
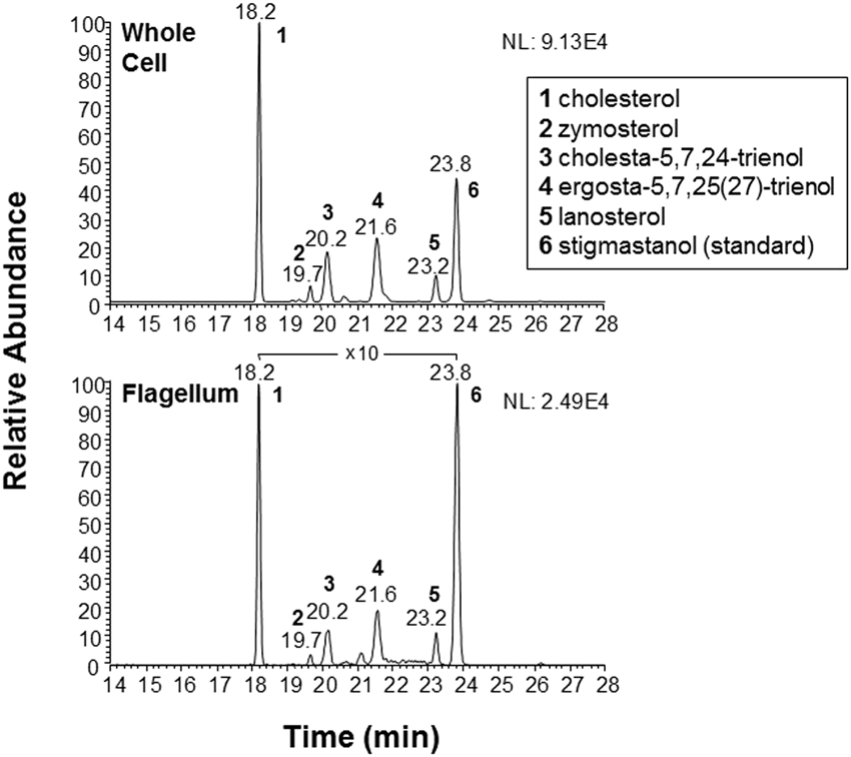
Sterol analysis by GC-MS of whole cell and flagellum extracts of procyclic *T. brucei* cells. Base-peak chromatograms of whole cell (top) and flagellar (bottom) extracts are shown. Peaks: **1**, cholesterol; **2**, zymosterol; **3**, cholesta-5,7,24-trienol; **4**, ergosta-5,7,25(27)-trienol; **5**, lanosterol; **6**, stigmastanol (internal standard). Base-peak chromatograms were plotted based on the quantitative ion specific for each sterol ion species in positive-ion mode, as follows: mass to charge ratio (*m/z*) 368 (cholesterol), 351 (zymosterol), 349 (cholesta-5,7,24-trienol), 363 (ergosta-5,7,25(27)-trienol), 393 (lanosterol), and 383 (stigmastanol). 10X indicates magnification of the chromatogram. See Supplementary Fig. 1 for total ion spectra of each of these species.

Analysis of whole cell and flagellar sterols by gas chromatography-mass spectrometry (GC-MS) using stigmastanol as an internal standard revealed the concentrations of individual sterol species to be similar in the flagellum and whole cell (Fig. 2). Because PFR2 is only found in the flagellum, it was possible to use the western blot result of Fig. 1 to determine the number of flagella/equivalents in each sample and use this information to calculate cell body sterol levels by subtracting the flagellar sterol levels from the whole cell sterol levels (Table 1; see Methods for details). We normalized the sterol amounts to the protein concentrations in the samples, since the concentration of no single molecule has been accurately determined in separate cell compartments. However, since the protein concentration in flagella is much higher than in a cell body due to the scant amount of cilioplasm^47^, the actual enrichment is likely to be much higher. The individual sterols detected included cholesterol, zymosterol, cholesta-5,7,24-trienol, ergosta-5,7,25(27)-trienol and lanosterol. Among these sterols, enrichment of zymosterol was the lowest in the flagellum (1.4X) while ergosta-5,7,25(27)-trienol had the highest enrichment (5.2X). Most species found are derivatives of cholesterol or ergosterol, consistent with the previous work by the Nes group on the distribution of sterols in whole cells^41^. Importantly, although sterols are enriched in the flagellum, the relative amounts of each sterol species analyzed are similar in flagellum and whole cell fractions (Fig. 2). Cholesterol, the most abundant sterol, was significantly different from other sterols within whole cell and flagellar samples and between the two samples (p = 0.0005). A separate phospholipid analysis of flagellar and whole cell extracts was determined after solid-phase extraction and analysis by electrospray ionization-linear ion trap-mass spectrometry, in both negative- and positive-ion modes. The phospholipid profiles of these two extracts differed in both modes (data not shown). Because detailed phospholipid identification was performed by the Butikofer group^32^, we did not do this analysis here.

**Table 1:** Sterol composition of procyclic cell bodies and flagella, normalized to the protein concentration in the sample.

Peak	Sterol Species	Cell Body (pmol/mg protein) (relative abundance %)	Flagellum (pmol/mg protein) (relative abundance %)	P value**	Flagellum: Cell Body Ratio***
1	Cholesterol	1.45 ± 0.09* (47.6)	6.02 ± 0.34 (52.1)	0.0008	4.2
2	Zymosterol	0.14 ± 0.001 (4.6)	0.19 ± 0.006 (1.7)	0.13	1.4
3	Cholesta-5,7,24-trienol	0.72 ± 0.01 (23.5)	1.57 ± 0.03 (10.2)	0.006	2.2
4	Ergosta-5,7,25(27)-trienol	0.52 ± 0.05 (17.1)	2.69 ± 0.15 (22.4)	0.009	5.2
5	Lanosterol	0.22 ± 0.01 (7.3)	0.51 ± 0.05 (13.6)	0.013	2.3
	Total	3.04 ± 0.16 (100)	11.54 ± 0.58 (100)		3.8

*Mean ± SEM. **Student’s T-test. Paired comparison of sterol levels normalized by protein concentration from Cell Body and flagellum using two biological replicates and three technical replicates. ***Ratios when normalized to procyclin instead of protein concentration are (top to bottom) 6.8, 1.8, 2.9, 4.5, 4.0, 5.0.

### Flagellar membrane liquid order in different life cycle stages of *T. brucei* is differentially susceptible to alteration by sterol-specific agents

Although lipid rafts contain high concentrations of sterols, all membrane domains of the cell contain sterols as well. Because procyclic and bloodstream cells differ in sterol composition, with procyclics having significant ergosterol derivatives in addition to cholesterol, we wondered whether they would be affected differently by agents specific for ergosterol and cholesterol. We previously employed dehydroergosterol labeling, filipin staining and Laurdan microscopy to assess the cellular distribution of sterols and membrane liquid order in trypanosomes. Laurdan is a fluorescent dye with emission properties that vary depending on the liquid order of the membrane environment it is in ref. 48. The spectral shift of Laurdan emission from red-yellow in the liquid-ordered phase of the membrane (more rigid) toward green-blue in the disordered phase (more fluid) is quantified by the generalized polarization (GP) function. In the experiment presented here, we employed a modified version of Laurdan, C-Laurdan, which, by virtue of an additional carboxyl group, is more sensitive to membrane polarity and has higher water solubility^49^. C-Laurdan imaging was performed with and without treatment with sterol-specific drugs MBCD and Amphotericin B (Amp B), which respectively target cholesterol^50–52^ and ergosterol^53^. Both MBCD and Amp B have been used extensively in previous investigations of lipid rafts^51, 52, 54–57^.

**Figure 3:**
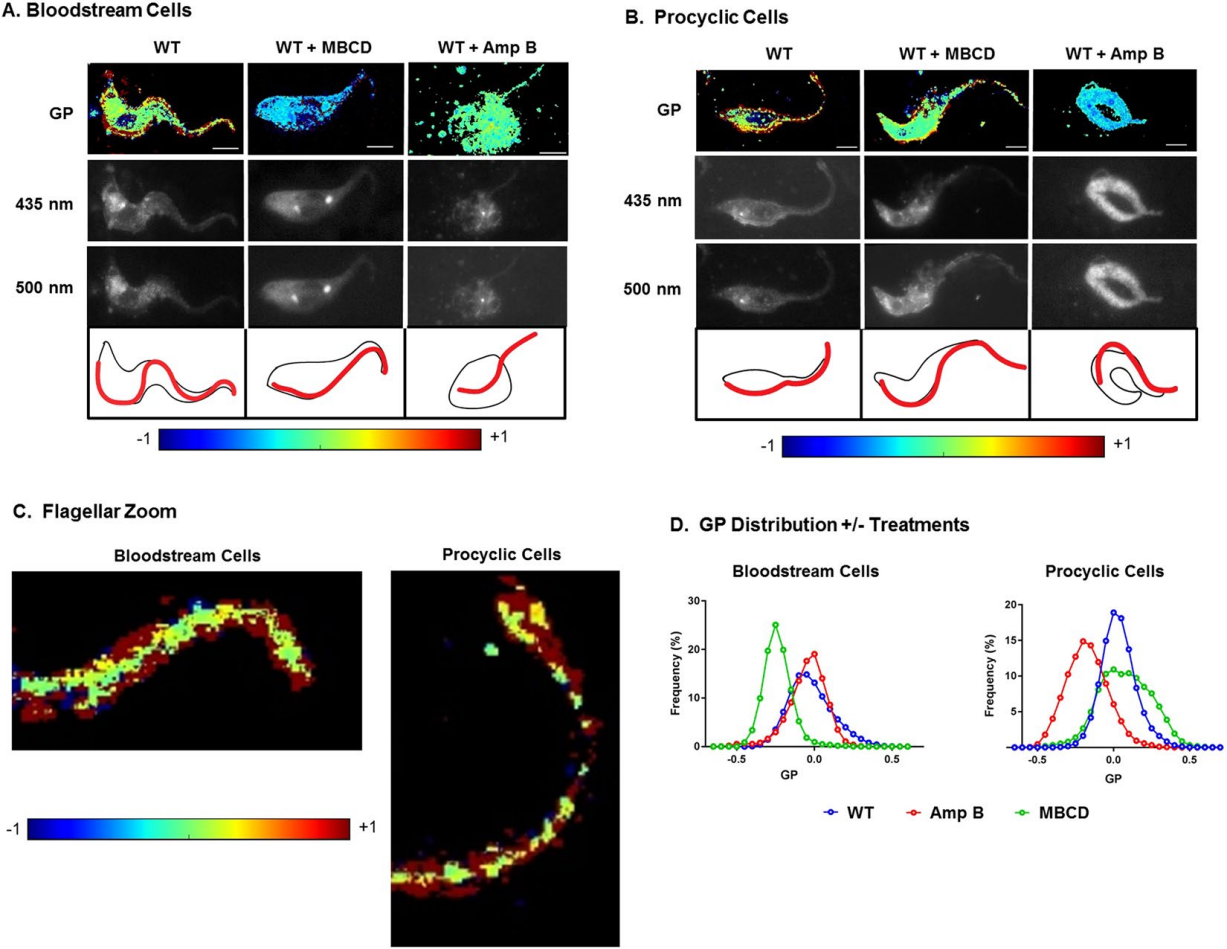
Sterol chelating agents disrupt membrane liquid order of bloodstream and procyclic cells differently. *T. brucei* bloodstream (**A**) and procyclic (**B**) cells were incubated with or without methyl-β-cyclodextrin (MBCD) or Amphotericin B (Amp B), stained with C-laurdan, and analyzed by fluorescence microscopy. Generalized polarization (GP) was calculated at each pixel in the two-dimensional image to determine the ratio of emission spectra obtained at 435 nm and 500 nm (see Methods). Higher liquid order (raft-enriched) is reflected by a higher GP (yellow-red), while lower liquid order is reflected by a lower GP (blue-green). The fluorescence images at the two wavelengths are shown in the middle panels and line drawings showing the cell body and flagellar membranes in black and red, respectively, are shown below these. Amp B treatment disrupts cell morphology but C-laurdan staining remains. MBCD has a milder effect on the cell morphology. The GP scale is provided at the bottom. Scale bar = 5 μm. (**C**) Higher power view of the anterior tips of untreated bloodstream and procyclic cells containing a thin cell body extension and mostly flagellum and having high GP. (**D**) Quantitative image analysis of individual pixels from cell images like those in (**A** and **B**) showing a reduction in liquid order in bloodstream cells by MBCD and in procyclic cells by Amp B from two biological replicates of procyclic cells and three biological replicates of bloodstream cells.

We obtained GP images of *T. brucei* procyclic and bloodstream cells stained with C-Laurdan (Fig. 3). The high membrane liquid order, reflected by yellow-red GP pixels, is most prominent in the flagellar membrane of both the bloodstream cells (Fig. 3A) and the procyclic cells (Fig. 3B), and is better appreciated in the higher magnification image (Fig. 3C). GP in bloodstream cells was reduced to below zero (blue) by treatment with cholesterol-targeting MBCD but was only slightly reduced by Amp B treatment (Fig. 3A). A strikingly opposite result was observed with procyclic cells (Fig. 3B), with MBCD having a modest effect on GP and Amp B reducing GP to below zero. The Amp B did disrupt the normal elongated cell morphology of both stages (right panels of Fig. 3A and B). To quantify observations in Fig. 3A–C, we generated a histogram distribution of individual GP pixel values across at least 10 cells from each stage, which clearly and quantitatively shows stage-specific negative (leftward) shift in GP by MBCD in bloodstream cells and by Amp B in procyclic cells (Fig. 3D).

### The lipid raft-dependent flagellar localization of calflagin is disrupted by sterol specific drugs

**Figure 4:**
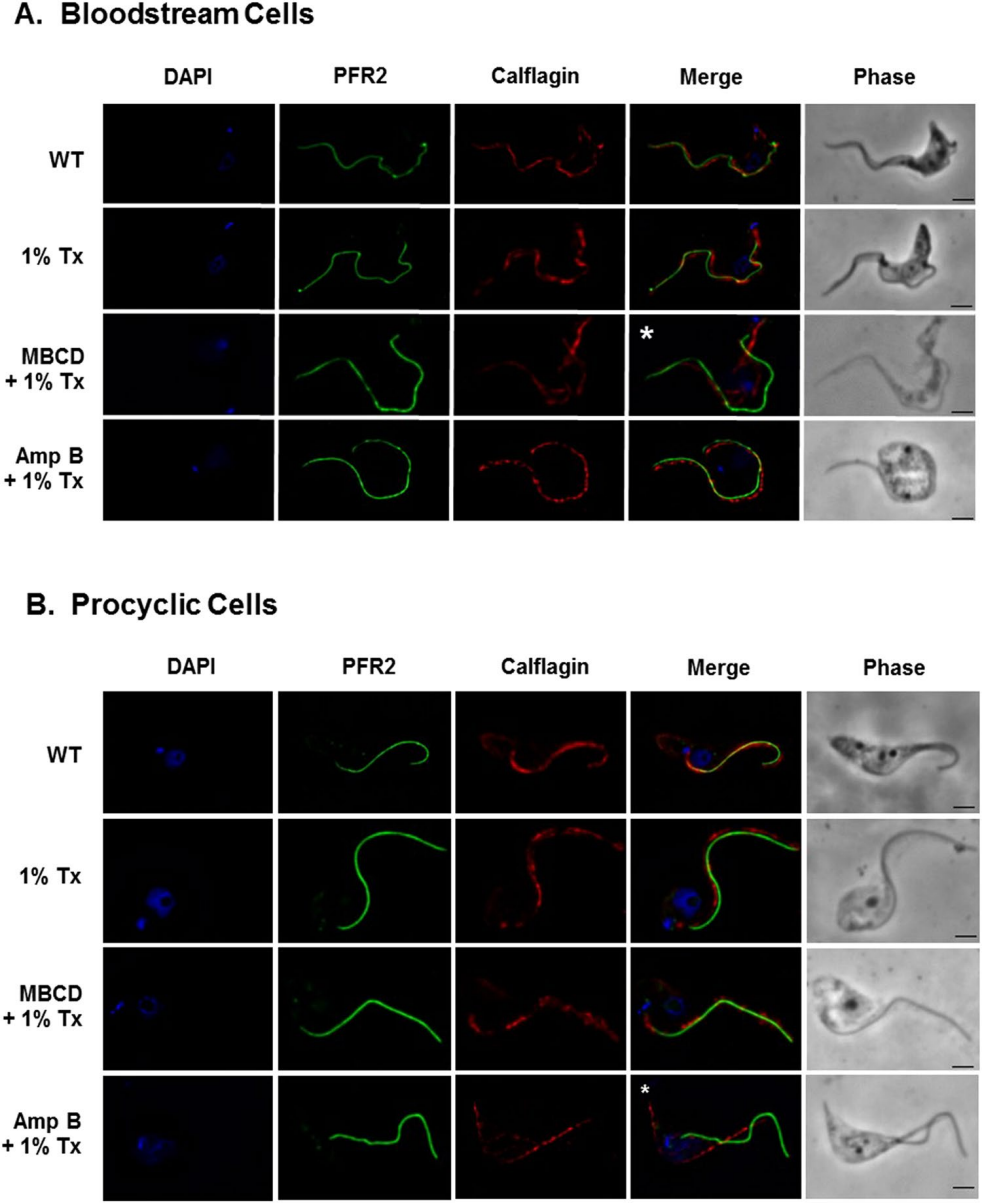
Sterol chelating agents disrupt localization of a dually-acylated flagellar membrane protein in bloodstream and procyclic cells differently. *T. brucei* (**A**) bloodstream cells and (**B**) procyclic cells were incubated with MBCD or Amp B, extracted with ice-cold 1% Triton X-100, fixed, and examined by immunofluorescence microscopy using antibodies specific for PFR2 (paraflagellar rod) or calflagin (dually-acylated flagellar membrane protein). Control WT cells were visualized only after fixation. Scale bar = 2 μm.

Calflagin is a myristoylated-palmitoylated calcium sensor located on the inner aspect of the *T. brucei* flagellar membrane. Its lipid raft association and flagellar membrane localization in bloodstream cells are both cholesterol-dependent and disrupted by MBCD treatment^9, 58^. To test whether MBCD and Amp B would disrupt calflagin localization differently in the two *T. brucei* life cycle stages, we examined calflagin localization in bloodstream cells and procyclic cells after treatment with these two drugs (Fig. 4). PFR2 is a flagellar cytoskeletal protein used as a control. Before imaging, cells were extracted with cold Triton X-100, which stabilizes lipid rafts due to their high liquid order^18^. MBCD but not Amp B disrupted calflagin localization in bloodstream cells (Fig. 4A), while Amp B but not MBCD disrupted calflagin localization in procyclic cells (Fig. 4B). Note the coincidence of PFR2 and calflagin staining in all merge panels other than those marked by asterisks in Fig. 4A,B.

### MBCD and Amp B disrupts of lipid rafts by altering sterols and not sphingolipids

**Figure 5:**
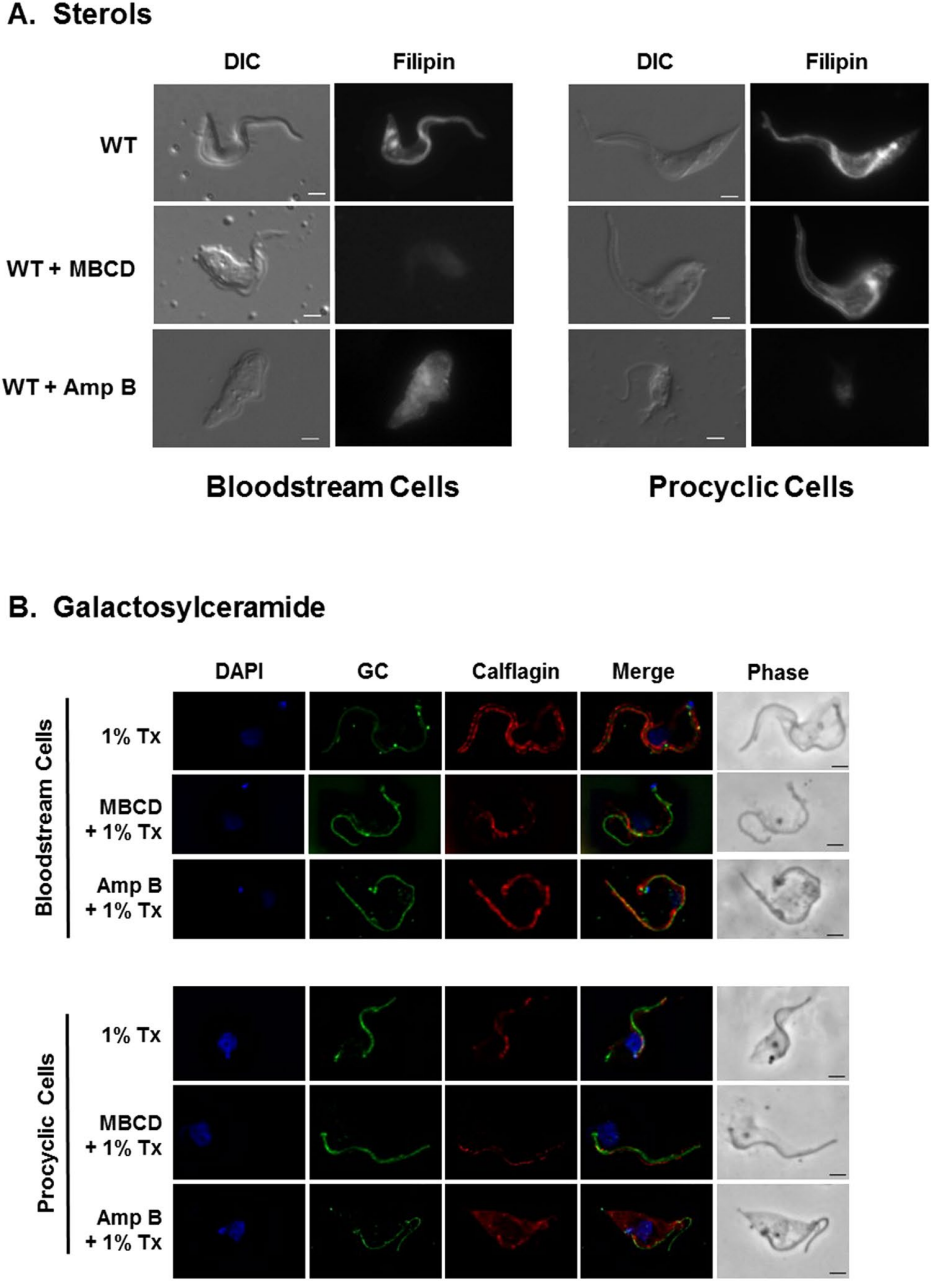
Sterol chelating agents remove sterols in bloodstream and procyclic cells differently. (**A**) *T. brucei* bloodstream cells and procyclic cells were stained with sterol-specific Filipin after pretreatment with or without MBCD or Amp B and visualized by fluorescence microscopy. (**B**) Cells were incubated with MBCD or Amp B, extracted with ice-cold 1% Triton X-100, fixed, and examined by immunofluorescence microscopy using antibodies specific for galactosylceramide (GC) and calflagin (dually-acylated flagellar membrane protein). Scale bar = 2 μm.

To test whether the lipid raft disruption by the sterol targeting drugs is sterol specific, we stained bloodstream and procyclic cells with filipin, a fluorescent polyene macrolide specific to sterols^59^ (Fig. 5A). MBCD treatment eliminated filipin staining in bloodstream cells but not procyclic cells, while Amp B eliminated filipin staining in procyclic cells but not bloodstream cells. By contrast, immunofluorescence microscopy for galactosylceramide (sphingolipid)^9, 60^ revealed no change upon treatment of either stage with either drug (Fig. 5B).

### Lipid raft association of calflagin is differentially affected in *T. brucei* life cycle stages by sterol-specific drugs

**Figure 6:**
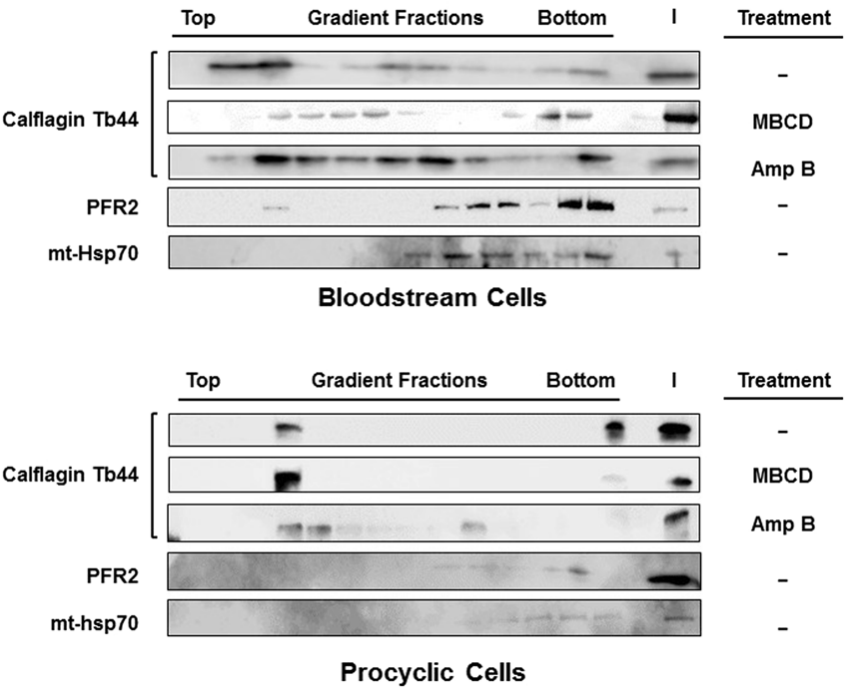
The lipid raft association of calflagin is affected differently by methyl-β-cyclodextrin (MBCD) and amphotericin B (Amp B) treatment of bloodstream and procyclic cells. Lipid raft extracts were prepared from *T. brucei* bloodstream and procyclic cells with or without pretreatment with MBCD or Amp B. Extracts were run on Optiprep gradients and gradient fractions were analyzed by western blotting using antibodies specific for calflagin (lipid raft) and PFR2 and mt-hsp70 (non-raft controls). I = input material loaded on the gradient.

Although the calflagin mislocalization induced by MBCD and Amp B treatments of bloodstream and procyclic cells indirectly implies a mechanism involving disruption of lipid rafts (Fig. 4), we directly tested this notion by lipid raft extraction^61^ and Optiprep gradient centrifugation^9^ (Fig. 6). Consistent with the findings of Fig. 4, the flotation (lipid raft association) of calflagin to the top of the gradient in bloodstream cells was affected more by MBCD than by Amp B (top), while calflagin flotation in procyclic cells was affected only by Amp B.

### Sterol specific drugs are differentially toxic to *T. brucei* bloodstream and procyclic cells

**Figure 7:**
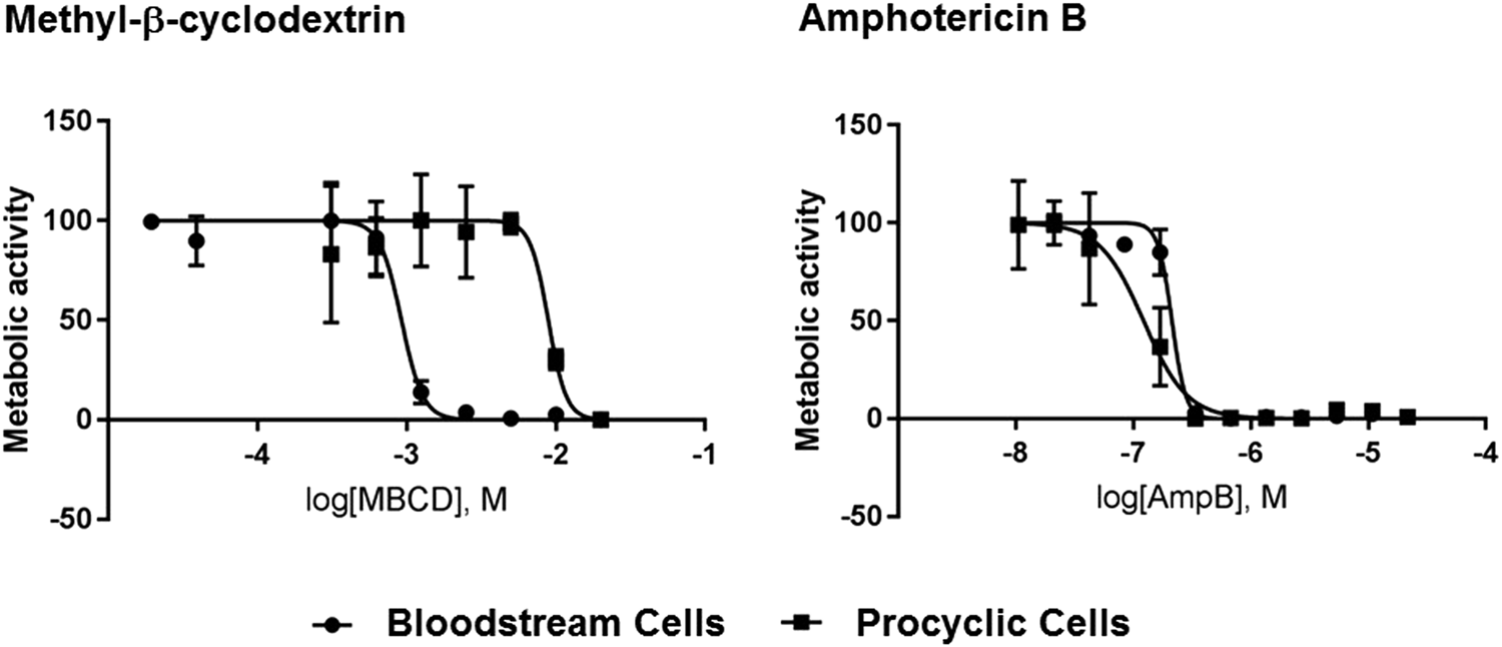
Bloodstream and procyclic cells are differentially susceptible to MBCD- and Amp B-mediated killing. *T. brucei* bloodstream and procyclic cells were cultured in the presence of different concentrations of methyl-β-cyclodextrin (MBCD) (20 μM to 20 mM) or Amphotericin B (Amp B) (1.32 nM to 43.2 μM) for 48 hrs. Metabolic activity was determined using a standard Alamar blue assay as described in Methods to generate IC_50_ curves. The IC_50_ values are given in Table 2. Error bars indicate SEM.

Given the stage specific differences in how Amp B and MBCD affect procyclic and bloodstream *T. brucei*, we investigated the cytotoxicity of these two drugs against the two life cycle stages using an Alamar-Blue cell viability assay (Fig. 7 and Table 2). Disruption or inhibition of synthesis of sterols has been a mainstay of antimicrobial chemotherapy against eukaryotic pathogens for over half a century^62, 63^. Amp B displayed an IC_50_ of 148 nM in procyclic cells, a three-fold lower value than the 422 nM for bloodstream cells. MBCD showed an IC_50_ of 1.0 mM in bloodstream cells, a nine-fold lower value than the 8.6 mM in procyclic cells.

**Table 2:** IC_50_ values for procyclic and bloodstream cells treated with methyl-β-cyclodextrin or Amphotericin B.

Life Cycle Stage	MBCD IC_50_ (mM)	AmpB IC_50_ (nM)
Procyclic Cells	8.6	148
Bloodstream Cells	1.0	422
**Procyclic:Bloodstream IC**_**50**_ **Ratio**	**8.51**	**0.351**

## Discussion

Lipids play important roles in eukaryotic cell membrane architecture, signal transduction and protein trafficking. Previously we used MBCD treatment of *T. brucei* bloodstream cells to determine the requirement of sterols (cholesterol) for calflagin targeting to the flagellar membrane, lipid raft association, and formation of detergent-resistant axoneme-associated particles^9^. We initially tried procyclic cells but they were refractory to MBCD. We hypothesized that procyclic cells were refractory to MBCD because of the difference in sterols in the life cycle stages, which by then was well known^41, 64, 65^. Although procyclic cells synthesize ergosterol and its derivatives, they still contain significant cholesterol which they acquire via receptor-mediated uptake of LDL present in the medium used for culture or found in the tsetse gut after the fly takes a bloodmeal. Indeed, the sterol composition of procyclic cells can be modulated simply by changing the sterol present in culture medium^41^. Our conclusion that flagella contain high levels of sterols was confirmed by dehydroergosterol labeling studies, and other components of lipid rafts like sphingolipids were found to be enriched in the flagellum as well^9^.

Work in the Simons Lab revealed that vesicles having unique lipid composition are formed in the Golgi, implying different pathways for the trafficking of specialized vesicles to different cellular compartments^66, 67^. This led us to wonder whether the sterols enriched in the flagellar membrane might differ from those in the cell body membrane. Our analysis revealed that, while sterols were more concentrated in the flagellar membrane than the cell body membrane, the levels of individual species in the two compartments were nearly identical (Table 1) and very similar to a whole-cell sterol profile of procyclic cells determined previously^42^. Approximately 67% of the sterol was cholesterol and its metabolites, with approximately 27% being ergosta-5,7,25(27)-trienol. A recent study reported ergosterol to be a signaling molecule for cell proliferation in both the life cycle stages of *T. brucei*, rather than simply an element of membrane architecture^68^. Consistent with these findings, only trace levels of ergosterol were detected in our fractions.

Although sterols are more concentrated in the flagellar membrane, the finding that the relative amounts of individual sterol species are remarkably similar in the flagellum and cell body is striking, given that levels of individual phospholipid species differ in these two compartments. The Butikofer group found that the flagella and whole cells respectively contain 41% and 20% phosphatidylethanolamine, 37% and 65% phosphatidylcholine, 11.4% and 48% sphingomyelin and 1.8% and 5.1% phosphatidylinositol^32^. How these differences are established and maintained is an important question^69^.

We further explored the differences in the lipid rafts of *T. brucei* procyclic and bloodstream cells using the sterol targeting drugs MBCD and Amp B. The sterol in bloodstream cells is over 96% cholesterol^32, 33, 42^. The composition of sterols in procyclic cells cultured in medium containing 10% fetal bovine serum varies among published studies but is approximately 60% cholesterol and 15–18% *de novo* synthesized sterols cholesta-5,7,24-trienol and derivatives of ergosterol^32, 33, 42^. These parallel our findings and likely reflect the levels found in procyclic *T. brucei* in the tsetse midgut, since there is ample cholesterol from the blood in the midgut of this hematophagous fly^70, 71^. The ergosterol biosynthesis pathway in *T. brucei* is unconventional with a novel metabolic network, which could affect the composition of ergosterol metabolites present not just as precursors but also as components of the membrane^40^.

C-Laurdan imaging of membrane liquid order in bloodstream and procyclic cells reflected our previously-reported^9^ high liquid order of the flagellar membrane (Fig. 3C). This is the first time GP imaging has been done on bloodstream form *T. brucei*, allowing direct comparison of the two life cycle stages. Although we had previously found bloodstream and procyclic cells to be respectively sensitive and resistant to MBCD-mediated sterol depletion, we were curious about that result given the ample cholesterol we found in the procyclic membrane (Fig. 2), originally described by the Nes group^40, 41^. However, the procyclic cells are indeed resistant to MBCD (Fig. 3A,D). One explanation for this apparent dichotomy is that the presence of ergosta-5,7,24(25)-trienol confers resistance to MBCD. Ergosterol has a higher ordering effect on phospholipid acyl chains compared to cholesterol^72–74^ and ergosterol-containing lipid rafts are more rigid and broader than those composed of cholesterol^75^. Our comparison of sterols showed 5.2X enrichment of ergosta-5,7,24(25)-trienol in the flagellum compared to the cell body (Table 1), suggesting higher affinity and/or partitioning of this sterol to the flagellar membrane, which could contribute to this resistance.

Amp B, a metabolite of *Streptomyces nodosus*, is one of the oldest anti-fungal antibiotics with selectivity for membranes containing ergosterol^76^. Its primary mechanism of action is the removal of free ergosterol via a sponge-like mechanism^77^ and a secondary mechanism involves membrane permeabilization via channel formation in an ergosterol dependent manner^53, 55^ with higher affinity for lipid rafts because of the higher order^76, 78^. More recent studies indicate that Amp B can induce the production of reactive oxygen species^79^. A combination of these harsh effects of Amp B is most likely the reason it severely affects the cellular morphology of *T. brucei* cells in both the life cycle stages. Although Amp B incorporates into membranes containing cholesterol and ergosterol equally, the presence of ergosterol allows Amp B to exert structural changes on the membrane especially at lipid rafts^80^. Amp B increases the orientational order of membranes rich in cholesterol while it induces membrane disorder when ergosterol is present^81^. This explains the inability of Amp B to disrupt lipid rafts in the cholesterol-based bloodstream cells while simultaneously altering cell morphology (Fig. 3A). Consistent with the mechanism of action of Amp B, procyclic *T. brucei* is susceptible to this agent due to the presence of ergosterol metabolites in its membrane (Fig. 3B,C).

Since the functional groups that allow interaction between Amp B and ergosterol are identical in ergosterol-5,7,25(27)-trienol and ergosterol^53, 82^, we expected Amp B to bind similarly to ergosterol-5,7,25(27)-trienol and ergosterol. When treated with Amp B, the cellular morphology of both procyclic and bloodstream form *T. brucei* was severely affected. However, the liquid order remained high in the bloodstream form (Fig. 4A,C), while it was reduced in the procyclics (Fig. 4B,C). This suggests the presence of life cycle specific differences in lipid rafts of *T. brucei* determined by the presence of ergosterol isomers. One of the oldest studies comparing procyclic and bloodstream form *T. brucei* membrane by freeze-fracture electron microscopy showed the procyclic cell membrane to have increased levels of β-hydroxysterols compared to the bloodstream cell membrane^31^. Thus, lipid rafts in procyclic cells are most likely more ordered, broader and more stable than those in bloodstream cells. The axoneme-associated detergent-resistant membrane particles apparent by scanning electron microscopy are significantly larger in procyclic cells than in bloodstream cells^9^.

Although sterol specific filipin staining was affected, sphingolipid (galactosylceramide) localization was not affected by either MBCD or Amp B treatment in either of the life cycle stages (Fig. 5). Indeed direct inhibition of sphingolipid synthesis via treatment with an inhibitor of serine palmitoyltransferase or serine palmitoyltransferase RNA interference had no effect on calflagin localization, indicating that sphingolipids are not as important for lipid raft formation^83^. This is true of mammalian cells as well^84^. Galactosylceramide is a group of glycosphingolipids containing a terminal galactose residue (usually in the beta configuration) linked to a ceramide moiety, which can be very diverse in the fatty acid and in the long-chain base composition^85, 86^. These results are important since they speak to the specificity of the MBCD and Amp B treatments for sterols.

Having found that MBCD and Amp B disrupt membrane liquid order and calflagin localization to the ciliary membrane through removal of specific sterols in a stage-specific manner, we tested the effects of these agents on the lipid raft association of calflagin (Fig. 6). The results were consistent with the immunofluorescence assays—Amp B reduced the flotation of calflagin in the procyclic cells while MBCD had no effect. In bloodstream cells, however, MBCD reduced the flotation of calflagin, but so did Amp B, though to a lesser degree. Although the overall liquid order of bloodstream cells was not affected by Amp B (Fig. 3A,D), cell morphology was dramatically affected, and this could lead to alterations in protein-lipid raft association through a mechanism other than sterol chelation. Finally, we tested whether the stage-specific differences we observed with these two agents might be reflected in cell viability. We found that the IC_50_ of the two agents paralleled all of our previous experiments, with MBCD having a lower IC_50_ for bloodstream cells than for procyclic cells and Amp B having a lower IC_50_ for procyclic cells than for bloodstream cells (Fig. 7).

These findings and those of others indicate that consideration of life cycle stage-specific sterol composition of pathogenic protozoa is important for those developing new therapies based on sterol depletion. In the related protozoan parasites *T. cruzi* and *Leishmania*, endogenous ergosterols are much more abundant in the insect stages of those parasites. Unlike *T. brucei*, the sterol synthetic machinery of *T. cruzi* and *Leishmania* are highly active in the mammalian host, making ergosterol targeting with Amp B to be a treatment options. As in *T. brucei*, the sterol composition in *Leishmania* and *T. cruzi* can be altered by modifying the available sterol in the environment/medium^87, 88^. The presence of cholesta-5,7,24-trienol and ergosterol in the *L. donovani* membrane underlies the resistance of this organism to Amp B^89^. Similarly, the concentration of cholesta-5,7,24-trienol is increased in *T. cruzi* epimastigotes exposed to sterol biosynthesis inhibitors^90, 91^ and these cells are exquisitely sensitive to the ergosterol level for normal proliferation^92^. The higher level of cholesta-7,24-trienol and other ergosterol metabolites in *T. brucei* may be associated with resistance to anti-fungal drugs, given the extremely low levels of synthesized sterols especially in bloodstream cells^68^.

In this report, we describe the differences in lipid rafts of the two major life cycle stages of *T. brucei*, largely attributable to the unique chemistry of the raft sterols and the resulting stability of lipid raft domains. These differences between the bloodstream and procyclic stages result from a combination of regulation and/or rewiring of the sterol biosynthetic pathways and on the ability of the parasite to scavenge sterols and their precursors from its different environments. We found that different sterol species produce lipid raft domains having different biochemical and cellular properties and also confer differential sensitivity to drugs targeting sterols. This appears to be true of other digenetic parasites as well, so care should be taken when developing new agents against these pathogens. The enrichment of lipid rafts in the flagellum and the life stage-specific differences in sterol composition could imply the existence of stage-specific signaling that may underlie life cycle transitions in this and other parasites that move between insect and mammalian hosts. Since human ciliary defects, collectively referred to as the ciliopathies, underlie diseases of the kidney, respiratory tract and pancreas, developmental disorders, blindness, cancer, and a variety of congenital heart diseases^2, 93, 94^, the biophysical properties of the ciliary membrane are of special interest and can have major impact in medicine.

## Methods

### Cell culture

All *T. brucei* cells used for this study were derived from the procyclic 29–13 line or the bloodstream form Single Marker line^89^. Both were originally derived from Lister strain 427, antigenic type MITat1.2, clone 221a^90^, engineered to co-express bacteriophage T7 RNA polymerase and tet repressor to permit tetracycline-inducible transcription. Procyclic parasites were cultured at 27 °C in SDM-79 medium^91^ supplemented with 10% FBS (Sigma-Aldrich, St. Louis, MO), 7.5 μg/mL hemin, 100 U/mL penicillin/streptomycin, 50 μg/mL hygromycin, and 15 μg/mL G418. Bloodstream parasites were cultured at 37 °C with 5% CO_2_ in HMI-9 medium^91^ supplemented with 10% FBS, 10% serum plus medium complement (SAFC Biosciences, Lenexa, KS), 100 U/mL penicillin/streptomycin, and 2.5 μg/mL G418.

### Purification of flagella and analysis of purity

Flagella were purified from log phase 29–13 *T. brucei* cells with modifications to methods described previously^9, 92^. 3 × 10^10^ cells were collected by centrifugation at 1000 × g for 10 min and washed twice in Buffer A (5 mM Tricine, 0.04 mM EDTA, 1 mM MgCl_2_, 2.4 mM β-mercaptoethanol, pH 7.0). To shear flagella from the cell bodies, cells were resuspended at 3 × 10^8^ cells/ml in Buffer A containing 0.5 M sucrose and 0.1 mM CaCl_2_, and sonicated with two pulses of 10 seconds each at the 15% setting in a Branson Digital Sonifier (Danbury, CT). Cell bodies were removed from the Buffer A-sucrose-CaCl_2_ lysate by centrifugation at 1000 × g and the supernatant was saved. Residual flagella in the cell pellet were liberated by another cycle of resuspension-sonication-centrifugation. The two supernatants were combined and centrifuged at 2800 × g to remove remaining unlysed cells to yield the supernatant constituted a crude extract (CE) composed of flagella and cell lysate. This extract was applied to a discontinuous sucrose gradient (2.02 M, 1.84 M and 1.66 M sucrose) and subjected to ultracentrifugation at 130,000 × g for 4 hrs at 4 °C. Pure flagella (Flag) were collected from the interface of the 1.66 M and 1.84 M steps. A whole cell lysate (WCL) for western blotting was prepared by lysing 2 × 10^8^ cells in one mL of Laemmli buffer. Aliquots of the WCL, CE and Flag were used for microscopy and/or western blot analyses shown in Figs 1 and 2 and Table 1. The western blot allowed an estimation of the number of flagellum-equivalents in all three samples WCL, CE and Flag (Fig. 1), with CE and Flag samples prepared similarly using 100 μL aliquots of extracts and 5X Laemmli buffer. For immunofluorescence microscopy, slides were fixed for 30 min on ice in 4% paraformaldehyde prepared in PBS, and fixative was quenched by incubation with 50 mM glycine for 12 min. Slides were then incubated in blocking buffer (2% normal goat serum, 1% BSA in PBS) for 30 min and incubated overnight at 4 °C with α-tubulin-specific rabbit serum (Invitrogen, Carlsbad,CA) diluted 1:80, pellicular membrane marker whole cell body (WCB)-specific mouse serum (neat) and cell surface marker procyclin-specific mouse serum diluted 1:1000 (Cedarlane Labs, ON, Canada). Western blotting was performed using WCB- (1:50), procyclin- (1:1000), calflagin (1:1000), and PFR2- (1:2000) specific antibodies diluted and imaging was performed using a LI-COR Biosciences CCD Imager. Densitometry analyses on western blots were performed using Image Studio^TM^ Software. WCB antibody and PFR2 antibodies were kindly provided by Keith Gull and Kent Hill, respectively. Production of the calflagin antiserum was described previously^58^.

### Lipid extraction and mass spectrometry

All steps were performed in acid-washed glassware to avoid contamination with plasticizers, and all solvents and reagents were HPLC- or MS-grade (Thermo Fisher Scientific or Sigma-Aldrich). Lipid extractions were performed with a combination of modified Bligh and Dyer method^93^ and Folch’s partitioning^94^. Approximately 0.5 mg of protein from each of the whole cell and purified flagellum fractions were subjected to lipid extraction using chloroform:methanol:water (C:M:W, 10:20:8, v/v/v), vortexed for 2 min and centrifuged at 1,800 × g for 15 min at room temperature. The organic supernatant was saved and the procedure was repeated twice. The delipidated pellet was then extracted three times with C:M (2:1, v/v) and centrifuged at 1800 × g for 15 min. All C:M:W and C:M extracts were combined and dried under nitrogen gas stream. The resulting lipid extract was subjected to Folch partitioning using C:M:W (20:40:15, v/v/v) to obtain an upper phase, which contained mainly protein-free GPIs (glycoinositolphospholipids, GIPLs), and a lower phase, which consisted mainly of neutral lipids (sterols, sterol esters, and mono-, di-, and triacylglycerols), small glycolipids, and phospholipids. The Folch lower phase was further subjected to a solid phase extraction in silica gel to separate sterols from other classes of lipids^95^. Briefly, 100 mg silica gel (70–230 mesh, 60 Å, high purity grade, Sigma-Aldrich) were packed into borosilicate glass Pasteur pipettes (5¾″, Fisher Scientific) using Pyrex glass fiber wool (8-μm pore size, Sigma-Aldrich) as a sieve. The column was sequentially conditioned with 4 ml each methanol, acetone, and chloroform. Dried Folch lower phase samples from whole cell and flagellum extracts were redissolved in 3 ml chloroform and loaded onto the column. Lipids were sequentially eluted with 4 ml chloroform (neutral lipids), acetone (small glycolipids), and methanol (phospholipids and free fatty acids). Each fraction was collected into a 7-ml amber glass vial with PTFE-lined screw top (SUPELCO, Sigma-Aldrich). All samples were immediately dried under a constant flow of nitrogen stream and stored at −70 °C until use. Sterols eluted from the silica column in the chloroform fraction were derivatized using MSTFA (N-methyl-N-trimethylsilyltrifluoroacetamide) (Thermo TS-48910) at 50 °C for 30 min. Whole cell and flagellum samples were spiked with stigmastanol (0.05 mg/mL), as an internal standard, prior to derivatization. Trimethylsilyl (TMS) derivatives were analyzed by (GC-MS) in a Trace GC Polaris Q (Thermo Fisher Scientific, Waltham, MA), using a TR5-MS column (30 m × 250 mm × 60.25 mm, Thermo Fisher Scientific). The injector and ion-source temperatures were 250 °C and 200 °C, respectively. The initial column temperature was set to 170 °C for the first 3 min of each run, followed by a ramp of 20 °C/min, reaching a plateau of 280 °C, where this temperature was held for 17 min. Helium was used as carrier gas at a flow rate of 1.7 mL/min.

Samples were ionized in positive-ion mode and fragmentation of each ion species was performed by electron impact at 70 eV. Fragment spectra were collected at the 50–650 *m/z* range and analyzed by searching the spectral library (NIST library, available at Xcalibur^TM^ 1.4 Srl, Thermo Fisher Scientific). The identities of the major sterol species and the internal standard were confirmed by fragmentation (Supplementary Fig. 1A–F). Additionally, equimolar concentrations of cholesterol and stigmastanol were run on GC-MS and molar relative response factor (MRRF) calculated by computing the ratio of cholesterol standard area divided by area of stigmastanol standard area and used for the quantitation of each GC-MS peak obtained for whole cell and flagellum fractions.

### Calculation of cell body and ciliary sterol levels

Whole cell and flagellum samples were run in biological duplicate and technical triplicate. Xcalibur^TM^ software was used to compute baseline chromatograms for sterols and MRRF, using stigmastanol as an internal standard, to calculate the concentration of each sterol species present in a sample. Densitometry of the western blot of Fig. 1D was used to determine the number of flagella in the whole cell extract and flagellum equivalents in other extracts based on the amount of PFR2, which is quantitatively flagellum-specific. Raw sterol concentrations obtained post-MRRF calculations were first adjusted by the number of cells used for extraction in both whole cell and flagellar extracts. The cell body sterol amount was then obtained by subtracting flagellar sterol per unit cell from whole cell sterol per unit cell. Protein concentrations of whole cell and flagellar extracts were measured by BCA assay (Thermo Fisher Scientific) and used to normalize the amount of sterols in cell bodies and flagella.

### C-Laurdan microscopy and image processing

*T. brucei* bloodstream and procylic parasites were treated with 20 mM MBCD (powder directly added to the parasite culture), 20 μg/mL Amp B (20 mg/mL stock added at 1:1000 directly to the parasite culture) or no treatment for 30 minutes, collected by centrifugation at 1000 × g for 10 min, washed twice in PBS supplemented with 13 mM glucose (PBSG) and incubated with 5 μM C-Laurdan ((6-dodecanoyl-2-[N-methyl-N-(carboxymethyl)-amino]naphthalene), TP Probes®, South Korea) in PBSG for 40 min at 4 °C as described previously^42^. Cells were washed twice with PBSG and fixed on ice for 30 min using 4% paraformaldehyde. Cells were mounted and visualized immediately using the Deltavision OMX system on widefield setup (GE Healthcare, Little Chalfont, UK). Samples were excited with a 365/10 excitation filter and z-stack emissions for C-laurdan were collected simultaneously in the range of 415–455 nm and 490–530 nm. Generalized polarization (GP) calculations were obtained from images using equation for widefield Laurdan microscopy described previously^96^. The Python routine was used to convert a GP image to a binary image according to a threshold value that allows discriminating the cells from the background and assign a heatmap color based on the GP corresponding to every pixel of the two-dimensional image. Histograms were generated using Graphpad Prism Version 7.02 from the GP values with at least 10 cells analyzed for each combination of stage and treatment condition (10,000–46,000 pixels per cell).

### MBCD and Amp B treatment, detergent extraction and immunofluorescence microscopy

*T. brucei* bloodstream and procyclic cells were treated with 20 mM MBCD or 20 μg/mL Amp B or no treatment for 30 minutes, pelleted by centrifugation at 1000 × g for 10 min, washed twice in PBS supplemented with 13 mM glucose (PBSG), allowed to settle onto poly-L-lysine coated slides for 15 min and extracted with ice-cold 1% Triton X-100 on ice for 5 minutes. Slides were fixed for 30 min on ice in 4% paraformaldehyde prepared in PBS, and fixative was quenched with 50 mM glycine for 12 min. For immunofluorescence microscopy without detergent extraction, slides were fixed after PBSG washes and permeabilized with 0.2% Triton X-100 prepared in PBS for 5 min. Slides were incubated in blocking buffer (2% normal goat serum, 1% BSA in PBS) for 30 min and incubated overnight at 4 °C with calflagin-specific mouse serum diluted 1:500, PFR2-specific rabbit serum diluted 1:1000, or galactocerebroside (galactosylceramide)-specific rabbit serum (Sigma, St. Louis, MO) diluted 1:1000 in blocking buffer, followed by a 30-minute wash with PBS. Slides were then incubated for 1 hour in AlexaFluor488-conjugated goat anti-rabbit secondary antibody or AlexaFluor568-conjugated goat anti-mouse secondary antibody (Life Technologies, Carlsbad, CA) diluted at 1:500 in blocking buffer and washed for 30 min with PBS. After a quick wash with water, slides were allowed to dry and mounted in Prolong Gold Antifade Mountant with 6-diamidino-2-phenylindole (DAPI) (Life Technologies, Carlsbad, CA). Imaging was performed using a Zeiss AxioImager Z1 (Peabody, MA) using a 100X objective. Image acquisition and de-convolution were performed using AxioVision software (Peabody, MA).

### Filipin staining

*T. brucei* bloodstream and procyclic parasites were treated with 20 mM MBCD or 20 μg/mL Amp B or no treatment for 30 minutes, pelleted by centrifugation at 1000 × g for 10 min, washed twice in PBS supplemented with 13 mM glucose (PBSG), allowed to settle onto poly-L-lysine coated slides while being fixed with 4% paraformaldehyde prepared in PBS for 30 min on ice. Fixative was quenched with 50 mM glycine for 12 min. Cells were then stained with 0.1 mg/mL Filipin III (Life Technologies, Carlsbad, CA Sigma F4767) in PBS for 30 min followed by a 15 minute wash with PBS. Slides were allowed to dry and mounted in Gelvatol mount. Imaging was performed on a Zeiss AxioImager Z1 (Peabody, MA) using a 100X objective and UV filter set at 200 ms exposure. Image processing was performed using Axiovision software.

### Lipid raft isolation and western blot analysis

Lipid rafts were isolated using a detergent-free method^54^. 8 × 10^8^ cells of either 2913 procyclic or Single Marker *T. brucei*, with or without a 30-min treatment with 20 mM MBCD or 20 μg/mL Amp B, were pelleted by centrifugation at 1000 × g, washed twice with PBSG (PBS with 2.3 g/L of glucose) and resuspended in 1 mL of base buffer (20 mM Tris-HCl, pH 7.8, 250 mM sucrose) supplemented with 1 mM CaCl_2_, 1 mM MgCl_2_, and protease inhibitor. All the procedures that followed were carried out in a cold room. Cells were lysed by passage through a 22 gauge × 3 inch needle and centrifuged at 1000 × g. The supernatant was collected and the pellet was lysed again. The two supernatant fractions were combined and subjected to Optiprep^TM^ gradient (0–25%) ultracentrifugation as described previously^54^ at 52,000 × g using a SW41Ti rotor in a Beckman ultracentrifuge for 90 min. 800 μL fractions were analyzed by SDS-PAGE and western blotting with calflagin-specific mouse serum (1:1500), mt-hsp70-specific mouse serum (1:1000) or PFR2-specific rabbit serum (1:1000). Western blots were developed using methods described earlier.

### Alamar blue assay

Bloodstream and procyclic cells were left to grow on 96 well plate for 24 hrs before treating with either Amp B (concentrations ranging from 1.32 nM to 43.2 μM) or MBCD (concentrations ranging from 20 mM to 20 μM) for 48 hrs. Cells were then incubated in 600 μM resazurin for two hrs before reading the fluorescence at 536 nm excitation and 588 nm emission. Each drug treatment was performed in triplicate with multiple biological replicates. Data analyses and IC_50_ calculations were performed in GraphPad Prism version 7.02. The IC_50_ values are given in Table 2.

### Statistical analysis

A two-tailed, paired Student’s T-test was performed to compute the difference in sterol quantities between samples normalized using protein levels in each sample. Two-way ANOVA followed by Tukey’s multiple comparisons test at α = 0.05 was used to assess the differences between total and individual sterols in cell body and flagellum samples.

## Electronic supplementary material

Supplementary Figure 1
