# Supplementary material for: Sterol targeting drugs reveal life cycle stage-specific differences in trypanosome lipid rafts
Source: Sci Rep. 2017 Aug 22;7:9105. doi: 10.1038/s41598-017-08770-9 (PMC5567337; doi:10.1038/s41598-017-08770-9)

# Sterol targeting drugs reveal life cycle stage-specific differences in trypanosome lipid rafts

Aabha I. Sharma[1,2], Cheryl L. Olson[1,2], João I. Mamede[3], Felipe Gazos-Lopes[5], Conrad L. Epting[1,4], Igor C. Almeida[5], David M. Engman[1,2,6]

Departments of [1]Pathology, [2]Microbiology-Immunology, [3]Cell and Molecular Biology and [4]Pediatrics, Northwestern University, Chicago, Illinois, USA. [5]Department of Biological Sciences, University of Texas at El Paso, El Paso, TX, USA. [6]Department of Pathology and Laboratory Medicine, Cedars-Sinai Medical Center, Los Angeles, CA, USA. Correspondence should be addressed to I.C.A. or D.M.E. (email: icalmeida@utep.edu or david.engman@csmc.edu).

**Supplementary Figure 1. GC-MS fragmentation spectra of sterol species from whole cell and flagellar extracts of procyclic *T. brucei* cells.** A-F, Cholesterol, zymosterol, cholesta-5-7-24-trienol, ergosta-5,7,25(27)-trienol, lanosterol, and stigmastanol (internal standard). $[M]^+$, molecular ion; OTMS, O-trimethylsilyl fragment; $CH_3$, methyl group.

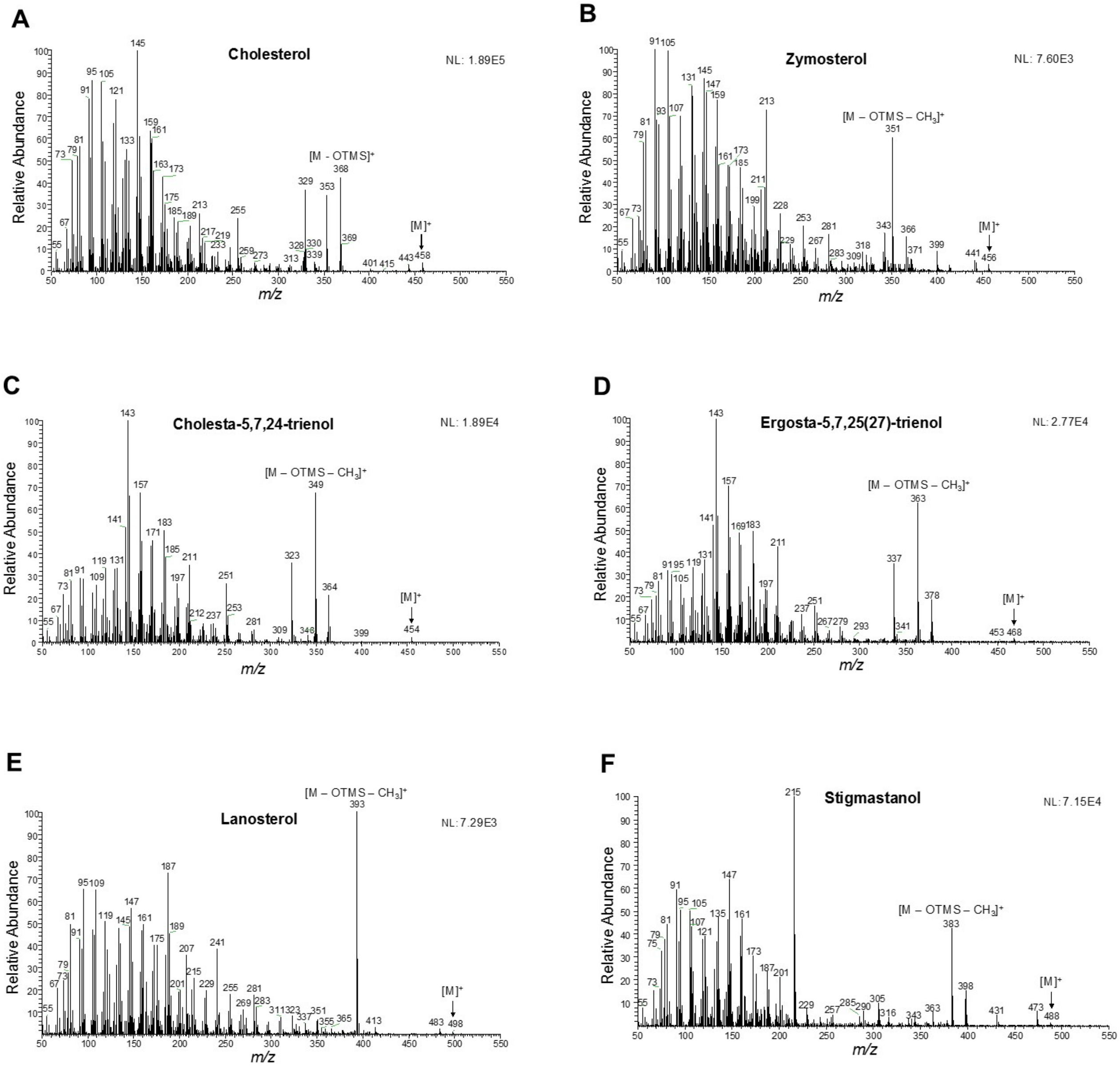

Supplement: Supplementary file 1 — Supplementary Figure 1 [file 41598_2017_8770_MOESM1_ESM.pdf]
